# Characterization of Polysaccharides from the Pericarp of *Zanthoxylum bungeanum* Maxim by Saccharide Mapping and Their Neuroprotective Effects

**DOI:** 10.3390/molecules28041813

**Published:** 2023-02-15

**Authors:** Mei-Bian Hu, Kui-Xu Gao, Yao Wang, Yu-Jie Liu

**Affiliations:** 1 Institute of Pharmaceutical & Food Engineering, Shanxi University of Chinese Medicine, Jinzhong 030619, China; 2 Key Laboratory of Traditional Chinese Medicine Processing of Shanxi Province, Shanxi University of Chinese Medicine, Jinzhong 030619, China

**Keywords:** *Zanthoxylum bungeanum* maxim, polysaccharides, oxidative stress, Nrf2/HO-1 signal pathway

## Abstract

The pericarp of *Zanthoxylum bungeanum* maxim (PZM) is a commonly used spice and herbal medicine in China. In the present study, the structural characteristics of PPZM were investigated by saccharide mapping after enzymatic digestion by using high-performance thin layer chromatography (HPTLC) and polysaccharide analysis by using carbohydrate gel electrophoresis (PACE). The mechanisms of protective effects of PPZM on Aβ_25–35_-induced oxidative damage were explored in PC12 cells. The results showed that PPZM contained 1,4-α-D-galactosidic, 1,4-α-D-galactosiduronic, and (1→4)-β-D-glucosidic linkages. Pretreatment with PPZM significantly increased the cell viability of Aβ_25–35_-injured PC12 cells. Flow cytometry and Hoechst/PI staining indicated that PPZM gradually relieved the apoptosis of the Aβ_25–25_-treated cells. PPZM markedly decreased the ROS level of PC12 cells and suppressed Aβ_25–35_-induced oxidative stress by increasing the SOD level, and decreasing the level of MDA and LDH. The mRNA expressions of caspase-3 and Bax were significantly downregulated, and Bcl-2 expression was upregulated by treatment with PPZM. PPZM significantly increased the mRNA expression of Nrf2 and HO-1 in Aβ_25–35_ treated cells. The results indicated that PPZM alleviated apoptosis and oxidative stress induced by Aβ_25–25_ through the inhibition of mitochondrial dependent apoptosis and activation of Nrf2/HO-1 pathway. PPZM can be used as a potential protective agent against Aβ_25–25_-induced neurotoxicity.

## 1. Introduction

Neurodegenerative diseases caused by aging seriously affect human health and quality of life in modern society [1]. Alzheimer’s disease (AD) is a chronic neurodegenerative disease closely related to memory and cognitive impairment [2]. The pathological mechanism of AD is still unclear, and there is no satisfactory treatment plan at present [3]. The main pathological features related to AD include β-amyloid (Aβ) plaque, neurogenic fiber tangle and neuron loss [4]. Several hypotheses have been proposed to explain the causes of AD, including the cholinergic hypothesis, Aβ hypothesis and Tau protein hypothesis [5]. Despite continuous debate about the Aβ hypothesis, evidence supports Aβ plays a significant role in the pathogenesis of AD [6,7]. In addition, oxidative stress is an early event in the progression from normal aging to AD pathology, and is considered to be a key harmful factor of AD. Aβ and oxidative stress are linked to each other, because Aβ induces oxidative stress, and oxidative stress increases the Aβ deposition. Studies have shown that the gradual accumulation of oxidative damage for a long time will lead to the appearance of clinical and pathological AD symptoms, including Aβ deposition [8,9].

Excessive reactive oxygen species (ROS) can lead to cell apoptosis, and many fatal diseases are related to abnormal ROS. The brain is the most vulnerable to oxygen free radical corrosion, because its antioxidant content is relatively low and its metabolic capacity is high [10]. Extensive studies have proven that the production of ROS leads to the defect of antioxidant defense system, which plays a key role in the pathogenesis of AD. With the environment and risk factors of aging, the brain gradually becomes highly sensitive to oxidative stress [11]. The accumulation of ROS will consume the activity and content of some antioxidant enzymes, induce some lipid peroxidation products and cause cell apoptosis [12,13]. In addition, Aβ can induce neuroinflammation and cause activation of reactive astrocytes and microglial cells. These cells cluster around amyloid deposits and sustain oxidative stress, leading to neuronal degeneration [14]. These pathological changes of antioxidant system and related apoptosis reactions are believed to cause cognitive impairment and progression of AD [15].

The pericarp of *Zanthoxylum bungeanum* maxim. (PZM), called Huajiao in Chinese, is a common food additive and herbal medicine used throughout the long history of China [16]. PZM is used to treat colds, stomach and abdomen pains, diarrhea, and vomiting in traditional Chinese medicine [17]. Previous studies have shown that PZM has a potentially therapeutic effect on AD, the unsaturated fatty acid amides hydroxy-α-sanshool (HAS) from PZM could improve scopolamine-induced learning and memory impairments in rats [18] and possessed antioxidant effects in H_2_O_2_-stimulated PC12 cells [19]. The mechanisms of HAS on D-galactose/AlCl_3_-induced AD mice via Nrf2/HO-1 signaling pathways were verified in our previous study [20]. Polysaccharides are also important active components in PZM, and our previous research has reported the structural characteristics and antioxidant activity of polysaccharides (PPZM) from PZM [21]. However, the glycosidic linkages in PPZM were still unknown, and the neuroprotective mechanisms of PPZM on AD treatment were also unclear. Compared with the previous study, this study further understood the structural information of PPZM by using saccharide mapping, the neuroprotective effects of PPZM were further confirmed by an Aβ_25–35_-induced damage model in PC12 cells, and the potential neuroprotective mechanisms of PPZM on AD were further explored through the Nrf2/HO-1 signaling pathway.

## 2. Results

### 2.1. Saccharide Mapping Analysis of PPZM

**Figure 1:**
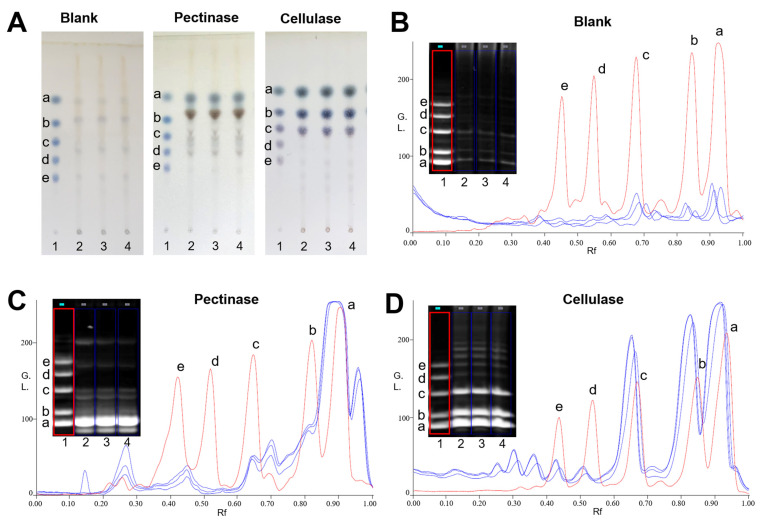
HPTLC profiles (**A**) and PACE fingerprints and chromatograms (**B**–**D**) of enzymatic hydrolysates of PPZM. Oligosaccharide standards (1), enzymatic hydrolysates of PPZM by corresponding enzymes (2–4). D-glucose (a), maltose (b), maltotriose (c), malttetraose (d) and maltpentose (e).

The saccharide mapping of PPZM after enzymatic digestion were obtained by HPTLC and PACE analysis. Pectinase and cellulase were selected for depolymerization of PPZM. As shown in Figure 1A, compared with samples before enzymatic hydrolysis, PPZM after pectinase digestion produced different small sugars (especially between D-glucose and maltose), which were obviously observed in HPTLC. The PACE analysis results of PPZM digested by pectinase was shown in Figure 1C, the resolution of PACE for enzymatic hydrolysis separation of PPZM was better than HPTLC. The chromatograms obtained by Gel-pro analyzer 4.0 software (Media Cybernetics, Bethesda, MD, USA) clearly showed the difference between enzymatic hydrolysis and samples before enzymatic hydrolysis. As shown in Figure 1A,D, different small sugars (mainly distributed between glucose and maltotriose) were also found in PPZM digested by cellulase, which were not found in samples before enzymatic hydrolysis. PPZM could be digested by pectinase and cellulase, indicating that PPZM contained 1,4-α-D-galactosidic, 1,4-α-D-galactosiduronic, and (1→4)-β-D-glucosidic linkages. 

### 2.2. PPZM Protected PC12 Cells from Aβ_25–35_ Induced Cytotoxicity

**Figure 2:**
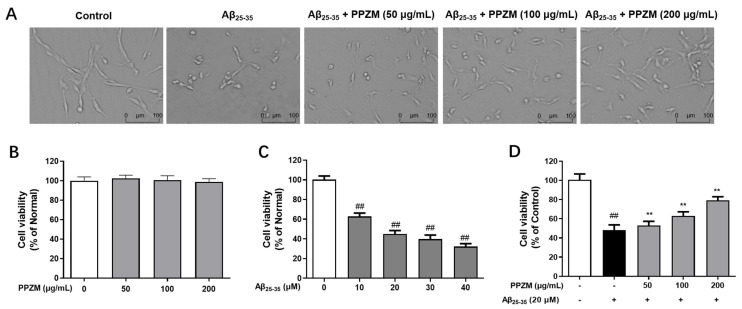
Neuroprotective effects of PPZM on Aβ_25–25_-induced neurotoxicity. (**A**) Cell morphology observed under a light microscope (the scale bar was 100 μm). (**B**) The effects of PPZM on the cell viability of normal PC12 cells. (**C**) The neurotoxicity of Aβ_25–25_ on PC12 cells. (**D**) The protective effects of PPZM on Aβ_25–25_ injured PC12 cells. Cell viability was measured by MTT. Results are presented as the mean ± SD. ^##^ *p* < 0.01 vs. the control group; ** *p* < 0.01 vs. the Aβ_25–25_ treated group.

As shown in Figure 2A, cells showed good cellular growth morphology in the control group without any treatment. However, after the incubation of Aβ_25–25_, the cell morphology changed, including the reduced cell quantity, membrane blebbing and cell shrinkage [22]. When pretreated with PPZM for 24 h, the morphology of the cells was observably improved, the above cell injuries alleviated. 

PPZM showed no cytotoxicity on normal PC12 cells at the tested concentrations of 50, 100, and 200 μg/mL (Figure 2B). Aβ_25–35_ (10–40 μM) significantly decreased the PC12 cell viability in a dose-dependent manner after 24 h of incubation (Figure 2C), and 10 μM dose was selected for further experiments. As shown in Figure 2D, pretreatment with PPZM (50, 100 and 200 μg/mL) significantly increased the cell viability of Aβ_25–35_ damaged PC12 cells (*p* < 0.01).

### 2.3. PPZM Inhibited Aβ_25–25_-Induced Apoptosis in PC12 Cells

**Figure 3:**
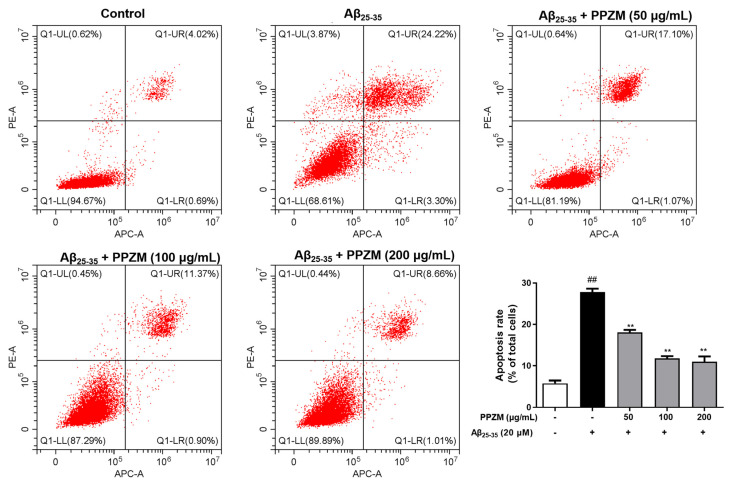
Effects of PPZM on Aβ_25–35_-induced PC12 cell apoptosis based on flow cytometry analysis. Results are presented as the mean ± SD. ^##^ *p* < 0.01 vs. the control group; ** *p* < 0.01 vs. the Aβ_25–25_ treated group.

**Figure 4:**
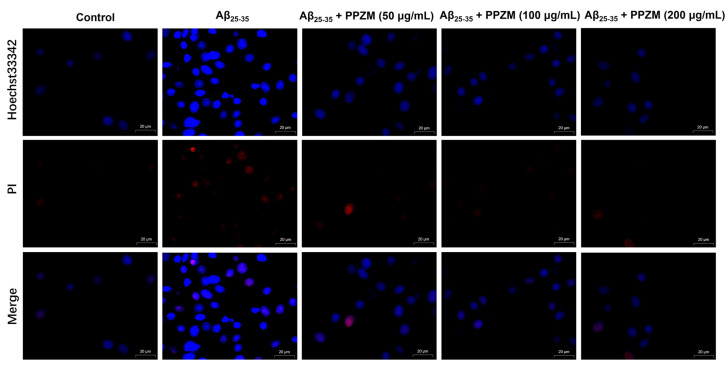
Effects of PPZM on Aβ_25–35_-induced PC12 cell apoptosis measured by Hoechst/PI staining. The morphological alterations were captured by laser confocal microscope. Scale bars were 20 μm.

Flow cytometry and Hoechst/PI staining were further used to determine the effect of PPZM on Aβ_25–25_-induced apoptosis in PC12 cells. As shown in Figure 3, the percentage of apoptotic cells and the total apoptosis rate were significantly increased after treatment with Aβ_25–35_, pretreatment with PPZM (50, 100, and 200 μg/mL) effectively decreased the apoptosis rate (*p* < 0.01), indicating that PPZM might inhibit Aβ_25–35_-induced cell apoptosis in PC12 cells. As can be seen in Figure 4, the control cells showed even fluorescence with the regular shape and uniform size of nucleus. The nuclear morphology appeared as condensed bodies and highly fluorescent in the Aβ_25–25_ treated cells. Pretreatment with PPZM (50, 100, and 200 μg/mL) gradually relieved the apoptosis of the Aβ_25–25_ treated cells in a concentration-dependent manner.

### 2.4. PPZM Suppresses Aβ_25–35_-Induced Oxidative Stress

**Figure 5:**
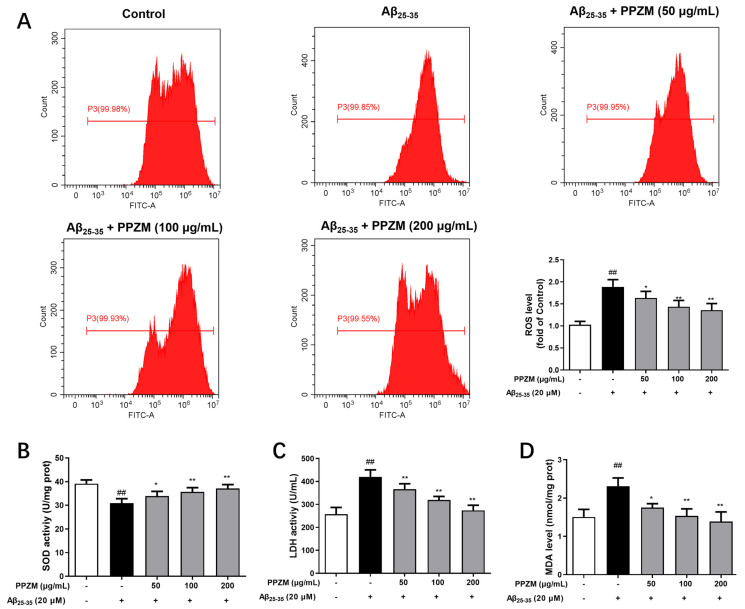
Effects of PPZM on Aβ_25–35_-induced oxidative stress in PC12 cells. (**A**) ROS level determined by DCFH-DA staining. The levels of SOD (**B**), LDH (**C**), and MDA (**D**) measured by using ELISA kits. Results are expressed as the mean ± SD. ^##^ *p* < 0.01 vs. the control group; * *p* < 0.05, ** *p* < 0.01 vs. the Aβ_25–25_ treated group.

ROS levels were measured to evaluate the effects of PPZM against oxidative stress by flow cytometry by using DCFH-DA staining. As shown in Figure 5A, compared with the normal cells, the ROS level significantly increased in the Aβ_25–35_-treated cells (*p* < 0 01). However, pretreatment of PPZM (50, 100 and 200 μg/mL) markedly decreased the ROS level in Aβ_25–35_ damaged PC12 cells (*p* < 0 05, *p* < 0 01, and *p* < 0 01). Effects of PPZM on the levels of SOD, LDH, and MDA in Aβ_25–35_-treated PC12 cells were further determined (Figure 5B–D). The level of SOD was significantly decreased, whereas levels of LDH and MDA were obviously increased in Aβ_25–35_-treated PC12 cells (*p* < 0.01). PPZM treatment significantly increased the SOD level, and decreased the level of MDA and LDH at all the tested the concentrations with concentration-dependent manners. 

### 2.5. PPZM Regulates the Expression of Apoptosis-Related and Nrf2/HO-1 mRNA

**Figure 6:**
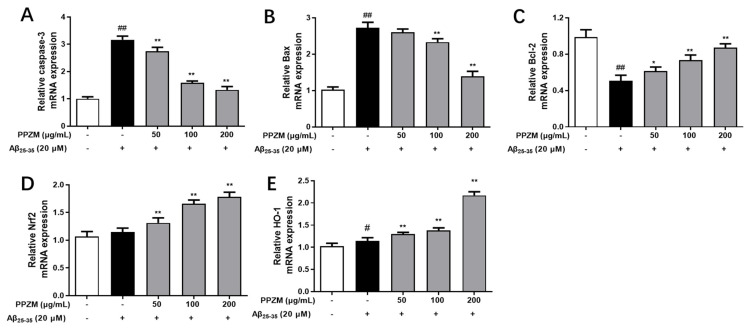
Effect of PPZM on Aβ_25–35_-induced apoptosis-related and Nrf2/HO-1 mRNA expression in PC12 cells. RT-qPCR analysis of caspase-3 (**A**), Bax (**B**) and Bcl-2 (**C**), Nrf2 (**D**), and HO-1 (**E**) mRNA expression in PC12 cells. Results are expressed as the mean ± SD. ^#^ *p* < 0.05, ^##^ *p* < 0.01 vs. the control group; * *p* < 0.05, ** *p* < 0.01 vs. the Aβ_25–25_ treated group.

Effects of PPZM on expressions of apoptosis-related caspase-3, Bcl-2 and Bax mRNA in Aβ_25–35_-treated PC12 cells were shown in Figure 6. As shown in Figure 6A–C, compared with the normal cells, mRNA expressions of caspase-3 and Bax were significantly upregulated, whereas Bcl-2 was markedly downregulated in Aβ_25–35_-treated PC12 cells (*p* < 0.01). PPZM treatment significantly decreased mRNA expressions of Caspase-3 and Bax, and increased the expression of Bcl-2 at the concentrations of 100 and 200 μg/mL (*p* < 0.01). The changes in expression of mRNA level of Nrf2 were also analyzed via RT-qPCR. As shown in Figure 6D, different concentrations of PPZM significantly increased the mRNA expression of Nrf2 (*p* < 0.01) compared with the Aβ_25–35_ treated PC12 cells. The expression of the downstream factor HO-1 in this pathway was further investigated. As the results shown in Figure 6E, PPZM significantly upregulated the mRNA expression of HO-1 in Aβ_25–35_ treated cells at the concentrations of 50, 100, and 200 μg/mL (*p* < 0.01). 

## 3. Materials and Methods

### 3.1. Chemicals and Reagents

Pectinase, cellulase, β-galactosidase, dextranase, β-mannase, and standards for maltose (purity ≥98%), maltotriose (purity ≥98%), malttetraose (purity ≥97%), and maltpentaose (purity ≥7%) were products of Beijing Solarbio Science & Technology Co., Ltd. (Beijing, China). D-glucose standard (purity ≥98%) was obtained from Sichuan Weikeqi Biotechnology Co., Ltd. (Chengdu, China). Moreover, 8-aminonaphthalene-1,3,6-trisulfonic acid (ANTS) was purchased from Meryer (Shanghai, China) Biochemical Technology Co., Ltd. (Shanghai, China). Aβ_25–35_ and dimethylsulfoxide (DMSO) were obtained from Sigma-Aldrich (St. Louis, MO, USA). Fetal bovine serum (FBS) was obtained from Gibco (Burlington, ON, Canada), Annexin V-APC/PI and Hoechst33342/PI dual staining kits were from Jiangsu KeyGEN BioTECH Co., Ltd. (Nanjing, China), Dulbecco’s modified Eagles medium (DMEM) cell culture medium was purchased from Procell Life Science &Technology Co., Ltd. (Wuhan, China). Trypsin-EDTA from Hyclone Laboratories (Logan, UT, USA). Detection kits for ROS, SOD, LDH and MDA were purchased from Nanjing Jiancheng Bioengineering Institute (Nanjing, China). TRIzol reagent and the RevertAid First Strand cDNA Synthesis Kit was purchased from Thermo Fisher (Waltham, MA, USA).

### 3.2. Polysaccharides Extraction

PZM were purchased from a local market in Hanyuan (ya’an, China). A specimen was stored at Shanxi Provincial Key Laboratory of Traditional Chinese Medicine Processing in Shanxi University of Chinese Medicine (Jinzhong, China). Polysaccharides from PZM were extracted by using the method in our previous report [21]. The obtained polysaccharides were redissolved and centrifuged (8000× *g*, 10 min), and then precipitated overnight by adding 95% ethanol (1:4, *v*/*v*). After being washed with anhydrous ethanol, acetone, and diethyl ether, the obtained polysaccharides (PPZM) were freeze-dried. 

### 3.3. Enzymatic Digestion of PPZM

PPZM water solutions (5 mg/mL, 0.5 mL) were added to certain enzymes (the final concentration of pectinase and cellulase were 20 and 10 U/mL, respectively) and digested for 16 h at 37 °C. Then the solutions were heated at 80 °C for 10 min to stop the enzymatic digestion. After centrifugation (4000× *g*, 10 min), the supernatants were dried by using a nitrogen evaporator at 40 °C. The PPZM solution without enzyme digestion was served as blank control.

### 3.4. Derivatization with ANTS

The derivatization was performed by using the reported method with some modifications [23]. Briefly, ANTS was dissolved in acetic acid/water (3:17, *v*/*v*) to prepare a solution of 0.1 mol/L. NaCNBH_3_ was prepared in DMSO (1 mol/L). Each dry enzymatic hydrolysate was added 125 μL of ANTS solution and 125 μL of NaCNBH_3_ solution, respectively. The mixture was centrifuged and incubated at 37 °C for 17 h. Then, the solution was dried by using a nitrogen evaporator at 40 °C. The derivatized samples were resuspended in 0.5 mL of 25% glycerin solution and stored at −20 °C. 

### 3.5. HPTLC Analysis

Sample separation (5 μL) was performed on 5 cm × 10 cm silica gel 60 plates (Merck, Darmstadt, Germany). Plates were first developed to a distance of 90 mm with ethyl acetate/glacial acetic acid/water (2:2:1, *v*/*v*/*v*) as mobile phase at room temperature. After being dried in air, the plates were redeveloped to a distance of 95 mm with the same mobile phase. Sugars were colorized with aniline-diphenylamine-phosphoric acid solution by heating at 105 °C for 10 min, and photographed.

### 3.6. PACE Analysis

PACE was performed according to the reported method [24]. In brief, all the samples (3–6 μL) were separated by using a mini-P4 vertical slab gel electrophoresis apparatus (Ji’nan Jun Yi Biotechnology Co., Ltd., Ji’nan, China). For separation of enzymatic hydrolysates, electrophoresis of 34% (*w*/*v*) polyacrylamide in the resolving gel with 8% (*w*/*v*) polyacrylamide in stacking gel was used. The 0.1 mol/L Tris-boric (pH 8.2) solution was applied as the electrophoresis buffer. The samples were electrophoresed at 15 mA to move the sample to the front end of the gel (observed with 365 nm UV lamp). Gels were imaged by using a 5000 Pro II gel imaging system (Guangzhou Biolight Biotechnology Co., Ltd., Guangzhou, China) under UV 365 nm.

### 3.7. Cell Culture

Rat adrenal pheochromocytoma PC12 cells were obtained from Procell Life Science &Technology Co., Ltd. (Wuhan, Hubei, China). Cells were cultured in DMEM supplemented with 10% FBS and 1% antibiotics (penicillin/streptomycin) in a humidified atmosphere of 5% CO_2_ at 37 °C. The medium was changed every 2–3 days.

### 3.8. Treatment and Cell Viability

Cell viability assay was performed by using MTT assay [25]. PC12 cells (5 × 10^3^/well) were seeded into 96-well plates and cultured for 24 h. The medium was then replaced with PPZM at concentrations of 0, 50, 100, and 200 mg/mL. After being incubated for another 24 h, 10 μL MTT solution (the final concentration of 5 mg/mL) were added and incubated for 4 h. The culture medium was removed, and 150 mL DMSO was added. The absorbance at 490 nm was measured by a SpectraMax^®^ PLUS 384 microplate reader (Molecular Devices, Sunnyvale, CA, USA).

The Aβ_25–35_ was dissolved in ultrapure water to prepare a 1 mM stock solution. The solution was incubated at 37 °C for three days to induce aggregate before use. PC12 cells were treated with Aβ_25–35_ at different concentrations of 10, 20, 30, and 40 μM for 24 h to determine the neurotoxicity of Aβ_25–35_. For evaluation of the neuroprotective effects of PPZM on the Aβ_25–35_-induced PC12 cells, cells were pretreated with PPZM at concentrations of 50, 100, and 200 mg/mL for 24 h and then exposed to Aβ_25–35_ (20 μM) for 24 h. The cell viability was measured by using the MTT method as described above.

### 3.9. Flow Cytometry Analysis for Apoptosis and ROS

Cells were seeded into 6-well plates and incubated for 24 h. Then PPZM at final concentrations of 50, 100, and 200 μg/mL were added to the cells. After being cultured for 24 h, cells were treated with 20 μM Aβ_25–35_ for another 24 h. Cells were harvested and washed by using PBS and stained by the Annexin V-APC/PI kit (Jiangsu KeyGEN BioTECH Co., Ltd., Nanjing, China). Cell apoptosis was detected by using a CytoFLEX flow cytometer (Beckman, Krefeld, Germany). In addition, the DCFH-DA ROS kit (Nanjing Jiancheng Bioengineering Institute, Nanjing, China) was used to determine the intracellular ROS level by flow cytometry.

### 3.10. Cellular Apoptosis Analysis by Hoechst33342/PI Dual Staining

Cells (2 × 10^5^ cells/well) were plated into 6-well plates for 24 h and then incubated with PPZM (50, 100, and 200 μg/mL) for an additional 24 h. Then, the cells were incubated with 20 μM Aβ_25–35_ for 24 h. The Hoechst 33342 and PI fluorescent dye were added for 15 min, and the nuclear morphology was observed with a FV100 laser confocal microscope (Olympus, Tokyo, Japan).

### 3.11. Quantitative Real-Time Polymerase Chain Reaction (RT-qPCR) Assay

Total RNA of the PC12 cells was extracted by using TRIzol reagent. The purity and concentration of each RNA sample was determined by the OD value at 260 and 280 nm. RNA (2 μg) was reversely transcribed into cDNA by using the RevertAid First Strand cDNA Synthesis Kit (Thermo Fisher, Waltham, MA, USA). RT-qPCR analysis was performed by using an ABI StepOnePlus System (Applied Biosystems, Foster City, CA, USA). The mRNA data were normalized to GAPDH by using the 2^−ΔΔCT^ method.

### 3.12. Statistical Analysis

Data obtained in the experiments are presented as mean ± standard deviations (SD). Statistical comparisons between groups were made by one-way analysis of variance (ANOVA) by using GraphPad Prism 9 software (GraphPad Software Inc., La Jolla, CA, USA). *p* < 0.05 was regarded as statistically different.

## 4. Discussion

As a neurodegenerative disease, AD brings an enormous financial burden for individuals and the society [26]. Therefore, it is urgent to make significant progress in order to find effective treatment for these diseases. Natural products isolated from plants have attracted much attention due to their high efficiency and biosafety, and polysaccharides are one of them. Many studies have shown that polysaccharides exhibit neuroprotective effects through a variety of mechanisms [27,28]. Experiments have shown that polysaccharides and polysaccharide-rich extracts can exhibit neuroprotective effects by promoting neurite outgrowth, and through Nrf2/HO-1, PI3K/Akt, NF-κB, and MAPK signaling pathways, etc. [29]. The present study indicated that PPZM alleviated apoptosis and oxidative stress induced by Aβ_25–25_ through the inhibition of mitochondrial-dependent apoptosis and activation of Nrf2/HO-1 pathway. Although further experiments are needed in AD animal models to support the clinical application of PPZM, this study provides a new perspective for the therapeutic potential of PPZM in treating AD.

It is reported that the saccharide mapping based on enzymatic digestion combined with HPTLC and PACE was one of the effective methods for the analysis of monosaccharides and oligosaccharides derived from polysaccharides [30]. The resolution of oligosaccharide separation with PACE is higher than that of HPTLC, whereas the resolution of monosaccharide separation with HPTLC is better than that with PACE [31]. Therefore, saccharide mapping using PACE and HPTLC analysis was used for analysis of the enzymatic hydrolysates of PPZM. Pectinase can act on the 1,4-α-D-galactosidic and 1,4-α-D-galactosiduronic linkages of pectic galactan and polygalacturonan, respectively [32]. Cellulase, also known as endo-1,4-β-D-glucanase, can participate in the breakdown of β-1,4-glucosidic linkages [33]. Our previous studies have shown that PPZM consist of mannose, rhamnose, galacturonic acid, glucose, galactose, and arabinose [21]. Therefore, the results indicated that PPZM contained 1,4-α-D-galactosidic, 1,4-α-D-galactosiduronic, and (1→4)-β-D-glucosidic linkages.

AD is the most common neurodegenerative disease in elderly people. Although great progress has been made in the mechanisms and treatment of AD, it is still an incurable disease [1]. Aβ directly or indirectly acts as a prooxidant, causing mitochondrial dysfunction and subsequent ROS generation [34]. Many studies reported that using antioxidants against Aβ-induced oxidative stress damage is a promising strategy to prevent AD [8]. In this study, the protective effects and possible mechanisms of PPZM in Aβ_25–35_-damaged PC12 cells were investigated. The results revealed that PPZM could inhibit Aβ_25–35_-induced cell damage by inhibiting apoptosis and reducing oxidative stress. 

Apoptosis usually occurs in the process of aging. It exists as a stable defense mechanism to maintain the number of cells in the body [35]. Studies have shown that death receptor pathway and mitochondrial pathway are two main pathways related to apoptosis [36]. Previous research has also shown that Aβ_25–35_ can cause cytotoxicity of PC12 cells by inducing apoptosis [37,38,39]. In the present study, Aβ_25–25_-induced apoptosis and the intervention of PPZM in PC12 cells were evaluated by flow cytometry and Hoechst/PI staining. It was found that treatment with PPZM significantly attenuated Aβ_25–35_-induced apoptosis in PC12 cells.

Mitochondria are the main site of ROS production, and oxidative stress is another important reason for Aβ_25–25_-induced cytotoxicity. The increase of ROS level in cells may lead to further pathological changes in brain neurons, which may lead to cognitive dysfunction. Studies have shown that in AD, oxidative stress can interfere with the process of mitosis, destroy cell cycles, and lead to apoptosis. In this study, we found that PPZM can play an antiapoptotic role in Aβ_25–25_-injured PC12 cells. We speculate that this effect may be related to the elimination of ROS by PPZM. The degree of oxidative damage can be measured by the LDH level, ROS scavenging enzyme SOD, and the final product of lipid peroxidation MDA [40,41]. Therefore, The LDH, SOD, and MDA levels were determined in this study to verify our hypothesis. As a result, PPZM significantly decreased the level of LDH and MDA, and increased the level of SOD in Aβ_25–25_-injured PC12 cells. 

Mitochondria also play a key role in regulating the cell death pathway associated with Bcl-2 family protein members [42]. Bcl-2 is an antiapoptotic protein which inhibits apoptosis, while Bax is a proapoptotic protein that can induce apoptosis in neurons. Unbalanced Bax/Bcl-2 ratio can lead to increased mitochondrial membrane permeability and damaged mitochondrial integrity [43]. Cytochrome c is released from mitochondria to cytosol to activate caspase protein and lead to apoptosis [20]. In this study, PPZM treatment significantly increased the Bcl-2 mRNA expression, and decreased the mRNA expression of Bax; the Bax/Bcl-2 ratio was decreased in Aβ_25–25_-injured PC12 cells. In addition, the caspase-3 mRNA expression was also downregulated by PPZM. These results suggest that the protective effect of PPZM in Aβ_25–25_-injured PC12 cells may be related to the inhibition of mitochondrial-dependent apoptosis.

The Nrf2 pathway is a key pathway for cells to resist oxidation and maintain homeostasis [44]. Under normal physiological conditions, Nrf2 and Keap1 bind together to maintain a relative inhibition state. Under the condition of oxidative stress, Nrf2 released from Keap1 and entered the nucleus. Then Nrf2 combined with the antioxidant response element (ARE) to activate the expression of Nrf2 regulating genes and enhance the ability of cells to reduce oxidative stress [45,46]. Under the condition of oxidative stress, the lack or activation disorder of Nrf2 can increase intracellular ROS. Excessive active ROS will lead to oxidative damage of many molecules, including DNA, protein, unsaturated fatty acid, etc., leading to cell dysfunction, apoptosis, and even necrosis [47,48]. The antioxidant enzymes including nicotinamide adenine dinucleotide phosphate: quinine oxidoreductase-1 (NQO1), haemoxygenase-1 (HO-1), and SOD, etc. play an important role in protection of the cell from ROS damage [49,50]. In the present study, PPZM treatment markedly increased the mRNA expression of Nrf2 and HO-1. The results indicated that PPZM alleviated oxidative stress and apoptosis induced by Aβ_25–25_, which might be closely related to the Nrf2/HO-1 pathway.

This study demonstrated that PPZM protected PC12 cells against Aβ_25–35_-induced oxidative damage via inhibiting mitochondrial dependent apoptosis and activating Nrf2/HO-1 signal pathway. However, Western blot, antagonist or inhibitor, and molecular biological studies are needed to verify this pathway. In addition, more research can be done in the future for further application of PPZM in the treatment of AD. First, the role and mechanisms of PPZM in the treatment of AD need to be further verified through animal experiments by employing multiple models. Secondly, further separation and purification of PPZM should be done to obtain pure polysaccharides, and the structure of pure polysaccharides should be identified by a variety of technologies, such as mass spectrometry and nuclear magnetic resonance analysis. Lastly, the structure-activity relationship between the therapeutic effect of AD and the polysaccharides needs to be clarified. 

## 5. Conclusions

In this study, PPZM were found to contain 1,4-α-D-galactosidic, 1,4-α-D-galactosiduronic, and (1→4)-β-D-glucosidic linkages. We demonstrated that PPZM significantly attenuated Aβ_25–35_-induced apoptosis in PC12 cells by decreasing the Bax/Bcl-2 ratio and downregulated caspase-3 expression. PPZM significantly decreased the level of LDH and MDA, and increased the level of SOD to suppress Aβ_25–35_-induced oxidative stress in PC12 cells. In addition, PPZM treatment markedly increased the mRNA expression of Nrf2 and HO-1 in Aβ_25–25_-injured PC12 cells. The results suggested that the protective effect of PPZM in Aβ_25–25_-injured PC12 cells may be related to the inhibition of mitochondrial-dependent apoptosis and alleviation of oxidative stress through the Nrf2/HO-1 pathway. PPZM can be used as a potential protective agent against Aβ_25–25_-induced neurotoxicity.

## Data Availability

Not applicable.

## Sample Availability

Samples of the compounds are not available from the authors.
